# Respiratory syncytial virus outbreak in neonatal intensive care unit: Impact of infection control measures plus palivizumab use

**DOI:** 10.1186/2047-2994-1-16

**Published:** 2012-05-02

**Authors:** Camila de A Silva, Lívio Dias, Sandra R Baltieri, Tatiane T Rodrigues, Neusa Brandolise Takagi, Rosana Richtmann

**Affiliations:** 1 Infection Control, Hospital e Maternidade Santa Joana, Rua do Paraíso 432, São Paulo, SP, 04103-000, Brazil; 2 Neonatology, Hospital e Maternidade Santa Joana, Rua do Paraíso 432, São Paulo, SP, 04103-000, Brazil

**Keywords:** Respiratory syncytial virus, Outbreak, Palivizumab, Neonatal intensive care

## Abstract

**Background:**

The occurrence of a respiratory syncytial virus (RSV) outbreak in a Neonatal Intensive Care Unit (NICU) is related to unfavorable outcomes, as this infection can lead to respiratory distress and death in premature in infants. Report the successful control of an outbreak that occurred in April 2010 in a NICU.

**Methods:**

After the index case, of 18 premature infants placed in the same room 10 infants were infected. Of those 10, 6 developed mild to moderate respiratory symptoms, 4 persisted asymptomatic and no death occurred. Contact and respiratory precautions were rapidly initiated, the infants were cohorted in 3 different rooms and palivizumab was administered to all contacts.

**Results:**

The outbreak was controlled and no new cases were subsequently indentified.

**Conclusion:**

Standard infection control measures plus palivizumab prophylaxis were efficient in rapid control of the outbreak.

## Introduction

Respiratory syncytial Virus (RSV) is a single stranded RNA virus of the Paramyxoviridae family. A and B sub-types are involved in the majority of outbreaks; the A subtype in responsible for most of them [1].

RSV can cause respiratory symptoms in patients of all ages, but most cases occur in children under one year [2]. Special populations as premature infants born before 35 weeks of gestational age (GA), patients with underlying lung disease and patients with congenital heart disease are at risk of more morbidity and mortality from RSV infection [3].

Transmission is most commonly by direct contact, as the virus can remain for hours in surfaces and the hands of health care workers [4]. When the virus circulates in the general population, health care workers and visitors can bring RSV to neonatal units. Infected infants are important sources of infection of others and they can remain excreting virus for longer periods [5].

RSV outbreaks in NICU are expensive besides the increased morbidity and mortality [6]. Conventional infection control methods as hand hygiene and patient isolation in cohorts are recommended, but those procedures should be supplemented by the use of palivizumab, a monoclonal antibody directed to glycoprotein F, as an effective adjuvant to standard infection control measures [7-9]. The aim of this manuscript is to report that the combination of infection control measures with passive immunotherapy in a country were new antiviral agents is not easily available, succeeded in the rapid control of an outbreak in NICU.

## Methods

Our NICU is located at a Private Maternity Hospital in São Paulo city with 1.000 births per month, has two NICU situated in different floors of the building and does not receive patients from other institutions. Each unit has an overall capacity of 25 beds distributed in a large hall with 21 beds and three in the isolation room. In addition, our hospital has one semi intensive care unit with 21 beds, distributed in 5 different rooms. The outbreak started in the NICU situated on the second floor.

Due to the seasonal characteristic of RSV in São Paulo (April to August), the palivizumab prophylaxis is indicated (intramuscular 15 mg/kg/day, each 30 days) to infants less than 32 weeks of GA, or congenital heart disease or chronic lung disease. The palivizumab antibody use depends on each patient health insurance authorization policy.

Routinely, the infant that presents respiratory symptoms suggesting viral infection is tested for RSV using an immunoassay test (**QuickVue RSV test – bio Mérieux**). Contact and droplet precautions with gowns, gloves and masks are promptly initiated in all suspect cases. The nasopharyngeal RSV test is repeated after 14 days of the diagnosis and then weekly to check the precautions measures necessity.

It is considered as RSV infection all neonates who present clinical evidence of lower respiratory tract infection and RSV immunoassay test positive. Neonate asymptomatic carriage was defined as a positive RSV immunoassay test and no clinical respiratory symptoms suggesting viral infection.

Protection rate of palivizumab was calculated by the number of contacts neonates free of RSV symptomatic disease who received palivizumab prophylaxis.

The suspicion of the present RSV outbreak began on 02 May 2010 as the first contact neonate of the index case presented clinical respiratory symptoms of viral disease.

## Results

The index case occurred in a 27-week GA, 965 g of birth weight preterm infant presenting on April 19^th^ tachypnoea, increase of respiratory secretions and respiratory failure requiring mechanical ventilation. The patient was 79 days old with bronchopulmonary dysplasia. The infant received palivizumab in the same day his rapid test became positive for RSV that means April 19th. At that moment, contact and droplet precaution were initiated. Two weeks apart the onset of RSV infection, the RSV test was still positive.

**Figure 1:**
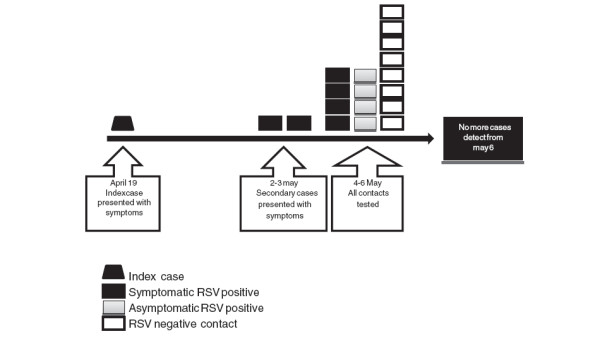
Summary of cases and contacts.

On 02 May/10 and 03 May/10 two patients in the same NICU of the index case developed respiratory symptoms and tested positive for RSV. Prevention control measures were then implemented and all infants in the same room were tested for RSV, even in the absence of respiratory symptoms. The cases and contacts babies were moved to a semi intensive unit to better cohort them into separate rooms as described below (Figure 1). Hand hygiene and environmental cleaning measures were reinforced.

All babies (18 infants) occupying the NICU by the moment of the outbreak were placed in 3 cohorts. Eight of them with negative RSV test were maintained for 7 days in a room under contact precaution considering RSV incubation period. None of these patients developed symptoms during this period and specific precautions were suspended. The remaining 10 infants were positive to RSV and they were placed into two different rooms according to the presence or absence of symptoms. Six presented symptoms and four were asymptomatic. All positive tested patients were isolated in incubators and the use of gloves and mask was oriented to visitors and staff personal.

All RSV positive infants were premature, average GA was 28-weeks (range: 25-34w), birth weight of 1181 grams (range: 560-2575 g) and 50 days old (range: 26–97) at RSV infection diagnosis. Patient characteristics are shown in Table 1.

**Table 1:** Characteristics of the patients in NICU with RSV positive test

	**GA (weeks)**	**Birth weight (grams)**	**Age (days) at RSV diagnose**	**Time of viral excretion**	**Underlying condition**	**Respiratory symptoms**	**Symptoms**
Index case	27	965 g	79	2 weeks	PDA/BPD	Yes - MV due to RSV infection	Yes
1	27	830 g	65	Negative control*	Hydrocelle/GI Malformation	YES – MV due to RSV infection	Yes
2	27	585 g	46	3 weeks	RDS/PDA/inguinal hernia	MV - Worsening of parameters	Yes
3	26	560 g	80	5 weeks	RDS/PDA/BPD	MV – worsening of parameters	Yes
4	33	1720 g	97	Negative control*	Onphalocele/PDA/PH/BPD	n-CPAP due to RSV	Yes
5	25	795 g	51	3 weeks	RDS/PDA/ARF	MV–worsening of parameter s	Yes
6	34	2575 g	38	3 weeks	RDS	Previous in MV	No
7	25	715 g	26	2 weeks	RDS/PDA	Previous in MV	No
8	25	650 g	26	2 weeks	RDS/PDA/BPD	MV – worsening of parameter s	Yes
9	27	1230 g	32	Negative control*	RDS/PDA	No – No 02 Support	No
10	32	2150 g	37	2 weeks	RDS	No – No 02 support	No

GA = Gestational Age; PDA = patent ductus arteriosus; BD = Bronchopulmonary dysplasia; RDS = Respiratory distress syndrome; GI = Gastrointestinal; PH = Pulmonary hypertension; ARF = acute renal failure; MV = Mechanical ventilation; n-CPAP: nasal continuous positive airway pressure.

*Negative Control – negative test on the 14th day after diagnostic.

Symptomatic infants presented mild to moderate symptoms, with cough, fever and coryza. Two patients (cases 1, 4) and the index case, however, required ventilation support due to RSV infection. Cases 3, 5 and 8 were already under mechanical ventilation and required an increase on ventilatory parameters after the infection. Cases 6, 7, 9 and 10 did not present symptoms. No specific antiviral treatment for RSV infection was given, except palivizumab. In Brazil we do not have inhalatory ribavirin avaible. No death occurred due to RSV infection.

All contacts and the index case received palivizumab. Cases 1, 2, 3 and 9, received palivizumab previously to the onset of outbreak according to institutional policy although cases 1, 2 and 3, presented respiratory symptoms of RSV infection. Palivizumab was prescribed to all other 14 patients occupying the same NICU within the beginning of the outbreak. Palivizumab protection rate for symptomatic infection was 67% (12/18 cases).

RSV positive cases were weekly tested after 14 days of the first detection and, in most cases, still positive at subsequent tests. The longest viral shedding time was 5 weeks. Symptomatic patients were isolated until hospital discharge and the asymptomatic ones were removed from the semi intensive unite when the viral test becomes negative. There were no new cases detected and the outbreak was successfully controlled in 5 days (onset 2 May/10 and last case 6 May/10).

## Discussion

Besides index case, ten RSV cases were diagnosed in our NICU, most of them under 28 weeks GA. Premature infants are highly susceptible to RSV infection due to the immaturity of their immunological system and the low levels of maternal antibodies [10]. The occurrence of RSV outbreak in NICU may be related to unfavorable outcomes, including death [11,12]. Fortunately, among patients included in this study no deaths occurred and only 30% of the infected patients needed ventilation support attributed to the infection.

Viral infection diagnosis is usually based on symptoms, but, in an NICU outbreak the investigation of all contacts is fundamental because of the possibility of asymptomatic infection. Therefore asymptomatic and symptomatic infants must be quickly identified and isolated to avoid viral dissemination. Contact precautions are well established as a control measure to RSV outbreaks and the use of masks might be added when the risk of respiratory particles dispersion is expected[13]. We incorporated the use mask by visitors and staff in order to prevent contamination and avoid carriers into the NICU. It is recommended that infected patients should be kept in cohorts away from the other healthy prematures. Our strategy to transfer patients to other unit prevented the interdiction of the NICU and allowed the admission of other patients.

Usually, RSV shedding time last for 2 weeks, however, under specific conditions such as immunosuppressed hosts and preterm infants this period might be much longer [5]. In the present study RSV excretion was documented for five weeks, showing that viral testing, despite the role of detecting new cases, is crucial to determinate the duration of precautions. The “quick test” to diagnose RSV was chosen due to the simplicity of the method, low cost and fast results. Dizdar et al. demonstrated the use of a quick test as an efficient method in order to identify and control a RSV NICU outbreak [9].

Presently, palivizumab use is not formally indicated in outbreaks control. However, it may attenuate the gravity of clinical manifestations and help to control viral dissemination, according to some authors [7,9,11,14]. Despite the use of palivizumab, ten patients included in this study presented with infection, six of them symptomatic and four asymptomatic. We believe that palivizumab did not prevent infection, probably because the infants were already in the incubation period of RSV. The presented symptoms however were mild and no death occurred.

The control of RSV nosocomial outbreaks remain a challenge to medical staff and infection control practitioners mainly because many institutions do not have adequate isolation facilities. Early detection of cases, reinforcement of hand hygiene and contact precaution remains the most important measures to control outbreaks. Although no independent analyses of each measure could be done, we believe that the use of palivizumab, contact and droplet precautions and the periodic viral testing to determinate the suspension of precautions were crucial for the successful control of the outbreak.

## **Competing interests**

RR received grants for lectures from Abbott. The other authors declare that they have no competing interests.

## Authors’ contributions

SCA carried out the infection control measures, and drafted the manuscript; DL drafted the manuscript; BSR carried out the infection control measures; RTT carried out the infection control measures; TNB carried out the clinical care of the newborns; RR carried out the infection control measures, and drafted the manuscript. All authors read and approved the final manuscript.
